# Corneal clearance and central endothelial cell repopulation despite graft detachment after Descemet membrane endothelial keratoplasty

**DOI:** 10.3205/oc000103

**Published:** 2019-04-04

**Authors:** Maria Daravagka, Andrei Nestler, Peter Wiedemann, Christian Girbardt

**Affiliations:** 1 Department of Ophthalmology and Eye Hospital, University of Leipzig, Germany

**Keywords:** DMEK, corneal graft detachment, corneal clearance

## Abstract

**Objective:** Descemet membrane endothelial keratoplasty (DMEK) is the gold standard procedure for treatment of primary corneal endothelial disorders. Graft detachment is a frequent complication of DMEK, which often requires re-operation or re-bubbling. However, several cases of spontaneous corneal clearance despite graft detachment after DMEK have been reported. The underlying mechanisms of this phenomenon are poorly understood. We report three cases of corneal clearance after graft detachment in patients with Fuchs endothelial dystrophy and provide a review of the literature.

**Methods:** An 81-year-old and a 69-year-old phakic patient as well as a 56-year-old pseudophakic patient with Fuchs endothelial dystrophy underwent Triple-DMEK and DMEK, respectively. All three patients presented postoperatively with blurred vision due to an almost complete detachment of the graft, as shown by slit-lamp photography and anterior segment optical coherence tomography (OCT).

**Results:** Without additional intervention, gradual corneal clearance and presence of endothelial cells on the posterior recipient’s stroma were observed in all patients three months postoperatively. Increase in endothelial cell density, decrease in central corneal thickness (CCT), recovery of corneal transparency, and improvement of visual acuity were documented in all patients.

**Conclusions:** Our findings support the theory of corneal clearance after Descemet membrane endothelial transfer (DMET) (“free-floating” donor Descemet graft in the recipient anterior chamber after descemetorhexis). Further understanding on endothelial homeostasis might lead to innovative approaches in handling endothelial disorders.

## Introduction

Descemet membrane endothelial keratoplasty (DMEK) is a standardized technique, which is utilized for treatment of primary or/and secondary corneal endothelial disorders [1] such as Fuchs endothelial dystrophy, posterior polymorphous corneal dystrophy and pseudophakic bullous keratopathy. The technique is based on the replacement of the recipient’s Descemet’s membrane and corneal endothelium selectively by a healthy donor graft.

DMEK is described as a superior technique over other lamellar techniques like Descemet’s stripping automated endothelial keratoplasty (DSAEK) for treatment of corneal endothelial disorders [2], due to minimal alteration of the posterior corneal stroma involved. Several reports on DMEK reveal a best corrected visual acuity (BCVA) of 20/25 or better (>0.8) in 75% of cases, while six months postoperatively 22–47% of patients attained a BCVA of at least 20/20 together with an endothelial cell density of about 1,800–2,000 cells/mm² [3], [4].

A successful outcome of DMEK has been strongly associated with the adherence of the donor graft to the posterior surface of the corneal stroma [3], [5]. The rates of graft attachment and subsequently the overall positive outcomes of DMEK depend not only on donor graft characteristics but also on the surgeon’s experience [3], [5]. Detachment of the graft has been reported in up to 35–63% after DMEK, making it the most common complication of the procedure. Detachment can appear either as peripheral graft edge non-adherence or as more extensive defect [5], [6]. In 18–62% of patients with graft detachment, a re-bubbling process is necessary (once in 13%, twice in 5%, and three times in 1.3% of the eyes) [5], [6]. 

Remarkably, successful outcomes after DMEK, despite graft detachment, have also been reported. In this report, we present three cases of positive outcome, following late graft detachment, left without re-intervention and we attempt a comprehensive discussion of this condition by providing a review of the literature. 

## Case descriptions

An 81-year-old phakic female, a 69-years-old phakic male, and a 56-year-old pseudophakic female presented in our clinic with progressive deterioration of visual acuity, epiphora and photophobia of both eyes. 

In all patients, preoperative slit-lamp examination revealed corneal edema with extensive cornea guttata in both eyes, caused by Fuchs endothelial dystrophy. A best-corrected visual acuity (BCVA) of 4/20, 6/20 and 2/20 was measured in the worst eye of each patient, respectively. In all patients, reliable measurements of endothelial cell density by corneal specular microscopy could not be obtained due to the advanced stage of endothelial dysfunction. Central corneal thickness (CCT) was 687 µm, 606 µm, and 832 µm, respectively, as measured by Oculus Pentacam^®^ Scheimpflug camera.

The first two patients underwent Triple-DMEK (phacoemulsification and intraocular lens implantation combined with DMEK) while the third one underwent single DMEK as described by Melles and colleagues [3]. In all patients, an inferior peripheral Nd:YAG iridotomy was performed prior to surgery. Both endothelium and Descemet membrane were stripped off from the donor cornea immediately prior to the procedure. In recipient eyes, a descemetorhexis of about 9 mm in diameter was performed and the central portion of the endothelium with Descemet membrane was removed. Through a 3 mm clear corneal incision, the 8.5 mm diameter posterior lamellar corneal graft was inserted into the recipient anterior chamber through a glass injector, positioned in correct orientation onto the posterior stroma and secured by an air filling of the anterior chamber. No intraoperative complications occurred and all patients were asked to lie flat on their back postoperatively. The postoperative treatment included a combination of steroid (Dexamethasone), antibiotic (Ofloxacin), and miotic (Pilocarpine) eyedrops as well as an intravenous injection of Methylprednisolone 100 mg for the first three days after surgery. 

The first and the third patient were discharged on the fifth postoperative day without presentation of any complications and with attached graft as documented by anterior segment OCT. In the second patient, re-bubbling was necessary due to incomplete attachment of the graft. During the re-bubbling procedure, the graft was accidentally partially folded. This patient was discharged after absorption of the air bubble. 

**Figure 1:**
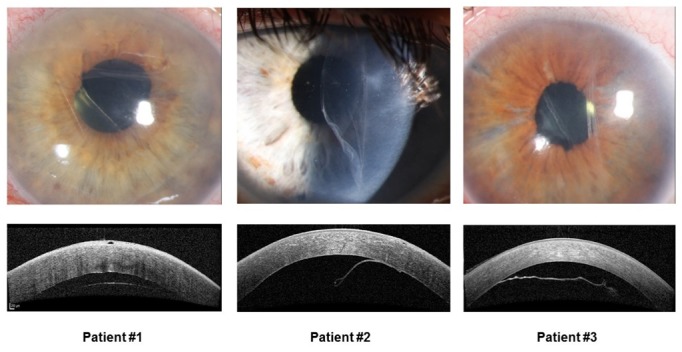
Postoperative corneal appearance and anterior segment OCT. In patient #1 and #3, the graft is largely detached, in patient #2 the graft is detached and partially folded.

Approximately one week following discharge, all three patients were referred to our clinic again with photophobia and increased blurry vision. Slit-lamp examination demonstrated diffuse corneal edema and corneal decompensation, highly likely as a result of graft detachment. Anterior segment OCT (Figure 1 (Fig. 1)) confirmed an almost complete detachment of the donor graft from the recipient’s stroma.

**Figure 2:**
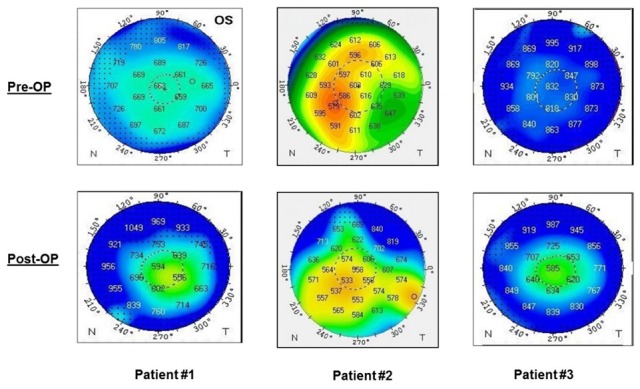
Comparison of central corneal thickness preoperatively and three months postoperatively demonstrating a decrease in thickness of the formerly swollen cornea in all patients

We followed a “wait & watch” approach in all three patients and close follow-up visits were undertaken. Remarkably, gradual corneal clearance was observed within three months in all patients. Upon slit lamp examination three months postoperatively, the corneal stroma was more transparent in all three patients, despite the fact that it was covered neither from donor tissue nor from the patient’s Descemet’s membrane. Central corneal thickness demonstrated a significant decrease from 687 to 572 µm (17%) in the first patient, a moderate decrease from 606 to 556 µm (8%) in the second one while in the third patient a more significant decrease from 832 to 580 µm (30%) was documented (Figure 2 (Fig. 2)). 

Those changes in corneal pachymetry were accompanied by an increase of visual acuity from 4/20 to 8/20 in the first patient, from 6/20 to 12/20 in the second and from 2/20 to 4/20 in the third one within the same time period. Although a marked improvement of the CCT was seen in all three cases, BCVA has not been a reliable outcome parameter. The reduced visual acuity despite the more advanced corneal clearance in the second and third patient was attributed to a concomitant retinal disorder and central retinal vein occlusion, respectively. Furthermore, the “free-floating” Descemet graft was positioned within the visual axis, causing blurry vision. 

**Figure 3:**
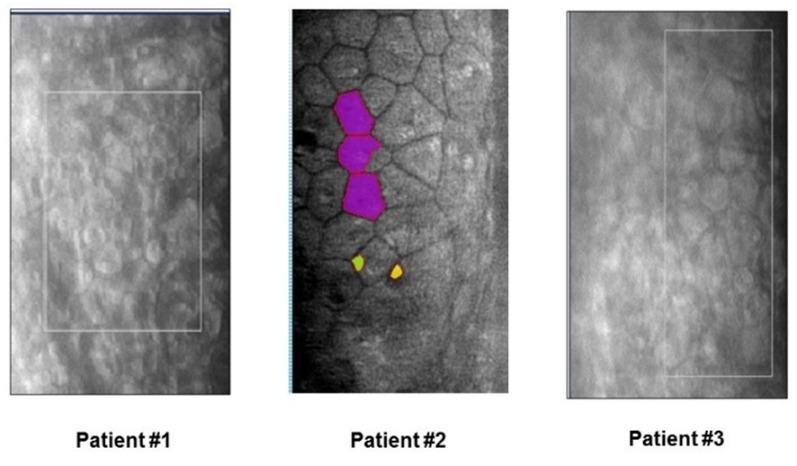
Noncontact corneal specular microscopy. Demonstration of endothelial cells on the posterior surface of the corneal stroma three months postoperatively, despite graft detachment

However, despite the detached donor tissue, enlarged and irregularly shaped endothelial cells were found by noncontact corneal specular microscopy on the recipient’s posterior corneal stroma in all patients (Figure 3 (Fig. 3)).

The above described positive findings were maintained over one-year follow-up, despite the still detached grafts. However, re-DMEK was performed after 14 months in the first patient, with successful graft attachment and a final visual acuity of 20/20. The second and third patient refused further interventions.

## Discussion

In this case report, we present three cases of spontaneous corneal clearance, improvement of BCVA and corneal re-endothelialization after almost complete graft detachment following DMEK, in three patients with Fuchs endothelial dystrophy. A decrease in central corneal thickness together with a presence of endothelial cells on the recipient’s posterior stroma was observed three months after the diagnosis of graft detachment without any additional operative intervention.

Similar to our observations, corneal transparency and improvement of BCVA along with presence of functional endothelial cells on the posterior stroma, despite graft detachment, have been previously reported. Of note, such positive outcomes were observed independently of the time of diagnosis and the extent of graft detachment. Dirisamer et al. described the involved patterns of corneal re-endothelialization following complicated DMEK, categorizing them in 4 groups depending on the extent of detachment and the positive or negative outcome [7]. Corneal clearance was reported in 26 cases, which included decentered, detached or even upside-positioned grafts. The presence of new healthy endothelial cells on the recipient’s corneal stroma despite non-adherence of the donor graft advocate that the graft endothelial corneal cells are able either to migrate onto the recipient’s stroma or to induce regeneration of the recipient’s peripheral endothelial corneal cells [7]. Interestingly, re-endothelialization of the recipient’s posterior stroma despite an immediate and complete graft detachment with “free floating” donor Descemet’s roll was also described by Dirisamer et al. [8]. The fact that the graft detachment occurred within few hours after DMEK and no contact of the donor graft with the recipient’s corneal stroma was observed indicates that the host’s endothelial cells from the peripheral cornea regenerate or graft’s endothelial cells migrate through the aqueous humour onto the recipient’s stroma [8], [9]. Additionally, in a prospective study, Dirisamer et al. demonstrated corneal clearance after Descemet membrane endothelial transfer (DMET), which describes a “free-floating” donor Descemet graft in the recipient anterior chamber after descemetorhexis [9]. They showed that DMET may be effective in the management of inherited endothelial “dystrophies”, particularly in Fuchs endothelial dystrophy but not in iatrogenic or surgical-induced “dysfunctions” referred to as bullous keratopathy [9]. Specifically, none of the eyes that underwent DMET for bullous keratopathy had a clinical improvement in corneal clarity. In contrast, all eyes with Fuchs endothelial dystrophy showed re-endothelialization and associated corneal clearance. 

Endothelial cell regeneration has also been discussed as a possible mechanism of re-endothelialization, found after a solely mini central stripping of Descemet’s membrane in patients with focal corneal edema without keratoplasty as well as in eyes with corneal graft detachment after DSAEK [10]. 

Jacobi et al. [11] addressed the origin of the new endothelial cells by investigating the areas of denuded corneal stroma between the donor graft and the recipient’s endothelium following successful DMEKs with *in vivo* confocal laser scanning microscopy. Comparison of the morphology of the endothelial cells between the denuded areas and the opposite or adjacent corneal quadrants revealed that the endothelial cells covering the patient’s denuded corneal stroma resembled those of the donor graft. This observation means that endothelial cells might migrate from the donor graft to the denuded corneal recipient’s stroma. However, this theory fails to explain the different response after DMET of patients with Fuchs endothelial dystrophy and bullous keratopathy.

Based on the above theories, we assume that the healthy endothelial cells seen in our patients are the result of either cell migration from the detached donor graft onto the posterior recipient’s stroma, regeneration of the remaining endothelial host’s cells, or a combination of both. The concept of regeneration allows the assumption that the endothelial cells themselves may not be primarily dystrophic but their dysfunction may be the result of specific conditions [12]. Presence of healthy endothelial cells, in this case the corneal graft’s cells, may trigger a regeneration process [9]. Such scenarios suggest that a simplified injection of endothelial cells into the anterior chamber, with or without keratoplasty, may prove equally efficient in managing endothelial disorders without the risk of complications associated with DMEK [13]. Alternatively, transplantation of cultured donor-derived corneal endothelial cells or differentiated stem cells may also provide a sufficient alternative treatment option for primary endothelial disorders in the future [14]. Surely, a perfect postoperative corneal anatomy as well as BCVA appears difficult to be obtained by a simple injection of endothelial cells without descemetorhexis [15]. However, the reported positive outcomes, including our current cases, consist a remarkable observation, which DMEK surgeons facing the complications of graft detachment should be aware of. A deeper understanding on the endothelial physiology and pathophysiology of the cornea in conjunction with information on the genetic background and stratification of the still undefined corneal endothelial disorders will contribute to the development of novel and safer treatment approaches. 

In conclusion, spontaneous recovery of corneal transparency along with improvement of visual acuity and increase in endothelial cell density is possible after graft detachment following DMEK, even without any further intervention. Such positive outcomes can be attributed to the increased endothelial cell repopulation by migration or/and by regeneration. These findings merit further study in regard with the corneal endothelial homeostasis in order to develop innovative approaches in handling endothelial disorders.

## Abbreviations

- BCVA: best-corrected visual acuity
- DMEK: Descemet membrane endothelial keratoplasty
- DMET: Descemet membrane endothelial transfer
- DSAEK: Descemet’s stripping automated endothelial keratoplasty
- CCT: central corneal thickness
- re-DMEK: repeat Descemet membrane endothelial keratoplasty
- Triple-DMEK: phacoemulsification and intraocular lens implantation combined with DMEK
- OCT: optical coherence tomography

## Notes

### Competing interests

The authors declare that they have no competing interests.

## References

[1] Bachmann B, Schaub F, Cursiefen C (2016). Therapie von Hornhautendothelerkrankungen mittels DMEK und UT-DSAEK. Indikationen, Komplikationen, Ergebnisse und Nachsorge. Ophthalmologe.

[2] Green M, Wilkins MR (2015). Comparison of Early Surgical Experience and Visual Outcomes of DSAEK and DMEK. Cornea.

[3] Dapena I, Moutsouris K, Droutsas K, Ham L, van Dijk K, Melles GR (2011). Standardized “no-touch” technique for descemet membrane endothelial keratoplasty. Arch Ophthalmol.

[4] Dirisamer M, van Dijk K, Dapena I, Ham L, Oganes O, Frank LE, Melles GR (2012). Prevention and management of graft detachment in descemet membrane endothelial keratoplasty. Arch Ophthalmol.

[5] Ang M, Wilkins MR, Mehta JS, Tan D (2016). Descemet membrane endothelial keratoplasty. Br J Ophthalmol.

[6] Spaniol K, Borrelli M, Holtmann C, Schrader S, Geerling G (2015). Komplikationen der Descemetmembran-Endothel-Keratoplastik. Ophthalmologe.

[7] Dirisamer M, Dapena I, Ham L, van Dijk K, Oganes O, Frank LE, van der Wees J, Melles GR (2011). Patterns of corneal endothelialization and corneal clearance after descemet membrane endothelial keratoplasty for fuchs endothelial dystrophy. Am J Ophthalmol.

[8] Dirisamer M, Ham L, Dapena I, van Dijk K, Melles GR (2012). Descemet membrane endothelial transfer: “free-floating” donor Descemet implantation as a potential alternative to “keratoplasty”. Cornea.

[9] Dirisamer M, Yeh RY, van Dijk K, Ham L, Dapena I, Melles GR (2012). Recipient endothelium may relate to corneal clearance in descemet membrane endothelial transfer. Am J Ophthalmol.

[10] Bleyen I, Saelens IE, van Dooren BT, van Rij G (2013). Spontaneous corneal clearing after Descemet’s stripping. Ophthalmology.

[11] Jacobi C, Zhivov A, Korbmacher J, Falke K, Guthoff R, Schlötzer-Schrehardt U, Cursiefen C, Kruse FE (2011). Evidence of endothelial cell migration after descemet membrane endothelial keratoplasty. Am J Ophthalmol.

[12] Bruinsma M, Tong CM, Melles GR (2013). What does the future hold for the treatment of Fuchs endothelial dystrophy; will ‘keratoplasty’ still be a valid procedure? Eye (Lond).

[13] Lam FC, Bruinsma M, Melles GR (2014). Descemet membrane endothelial transfer. Curr Opin Ophthalmol.

[14] Zavala J, López Jaime GR, Rodríguez Barrientos CA, Valdez-Garcia J (2013). Corneal endothelium: developmental strategies for regeneration. Eye (Lond).

[15] Birbal RS, Parker J, Dirisamer M, Janićijević A, Baydoun L, Dapena I, Melles GRJ (2018). Descemet Membrane Endothelial Transfer: Ultimate Outcome. Cornea.

[16] Baydoun L, van Dijk K, Dapena I, Musa FU, Liarakos VS, Ham L, Melles GR (2015). Repeat Descemet membrane endothelial keratoplasty after complicated primary Descemet membrane endothelial keratoplasty. Ophthalmology.

